# A Retrospective Study on the Prevalence and Surgical Management of Umbilical Infections in Calves in West Azerbaijan, Iran

**DOI:** 10.1155/vmi/1896579

**Published:** 2025-10-09

**Authors:** Razieh Torkaman, Saeed Azizi, Ghader Jalilzadeh-Amin

**Affiliations:** ^1^Department of Surgery and Diagnostic Imaging, Faculty of Veterinary Medicine, Urmia University, Urmia, Iran; ^2^Department of Surgery and Diagnostic Imaging, Faculty of Veterinary Medicine, Urmia University, Urmia, Iran; ^3^Department of Internal Medicine and Clinical Pathology, Faculty of Veterinary Medicine, Urmia University, Urmia, Iran

**Keywords:** calves, prevalence, surgical management, umbilical disorders, umbilical infections

## Abstract

**Objective:**

Umbilical infections are a prevalent worldwide health concern in calves. The prevalence and surgical management of umbilical infections in calves were investigated in this study.

**Animals:**

A total of 238 calves with umbilical infections.

**Methods:**

Medical records of 238 cases of umbilical infections in calves managed under either range conditions or in smallholder semi-industrial systems in West Azerbaijan, Iran, between March 1998 and July 2020 were analyzed. Data on husbandry type, breed, sex, age, and surgical outcomes were collected in this clinical retrospective study.

**Results:**

The prevalence of umbilical infections among calves was 8.66%. The frequency of infections in the external portion of the umbilicus was comparable to that of infections involving the intra-abdominal portion. Umbilical abscess was the most common infection in the external portion, while urachus infection predominated in the intra-abdominal portion of the umbilical remnants. Omphalitis was the least frequent infection observed in the newborns and omphaloarteritis was the least common pathology of the umbilical remnants. Calves under the range system had a significantly higher prevalence of umbilical infections compared to those in the semi-industrial system. The frequency of the infection was not affected by gender or breeds.

**Conclusion:**

Umbilical infection occurred with higher prevalence in calves under 13 weeks of age. The calves managed under range condition exhibited a higher prevalence of the infections with no breed- or gender-related differences. Surgical management was highly successful in treating the affected calves.

## 1. Introduction

Umbilical disorders, including umbilical hernias and umbilical infections, constitute a significant health concern among young calves, ranking as the third most common cause of disease in this demographic [1]. Umbilical swellings are frequently observed and can arise from hernia formation, umbilical infections, or a combination of both factors [2]. Umbilical infections, in particular, are prevalent worldwide among neonatal calves. The incidence of infection can be as low as 0% under well-ventilated, clean, and dry bedding conditions [3]. However, it could be increased to 9.0% [4] or 29.9% [5] based on the dairy farm husbandry and health conditions. Umbilical infections can potentially cause significant economic losses in dairy farms due to treatment costs, decreased growth performance, and potential mortality. However, the incidence and economic impact of the infection are not clear in Iranian dairy farms. Umbilical infections encompass various conditions such as omphalitis, umbilical abscess or chronic omphalitis, chronic active omphalitis, enlarged umbilical stalk abscesses, omphalophlebitis, omphaloarteritis, and infections or abscesses of the urachus [6, 7].

There exists a notable association between umbilical infections and an elevated risk of septicemia in neonatal calves [8]. Beyond local infection and inflammation, bacteria can disseminate hematogenously, leading to secondary infections in other organs. Consequently, septic arthritis may coincide with umbilical infections in certain cases [9]. Furthermore, calves affected by umbilical infections may concurrently be present with umbilical hernias [10]. A comprehensive diagnostic approach involving history, signalment, visual inspection, and meticulous physical examination, including palpation of both the external portion of the umbilical stump and deep palpation of the intra-abdominal umbilical cord portion in lateral recumbency, is typically appropriate for accurately diagnosing abnormalities of the umbilicus structures [7]. Clinical signs such as sinus/fistulae and moist purulent discharges further aid in the diagnosis of umbilical diseases. Additionally, the utility of transabdominal ultrasonographic assessment in providing reliable diagnostic support has been emphasized. This modality not only aids in diagnosing abnormalities of intra-abdominal umbilical remnants but also facilitates the selection of the optimal surgical approach for treatment [11].

Umbilical infections in calves can be managed through medical or surgical intervention; however, en bloc surgical resection of the infected umbilical remnant structures is generally recommended [2, 12–15]. Marsupialization of the umbilical vein has been documented as a treatment option for umbilical vein abscesses involving the liver [16]. Effective management of newborn calves includes maintaining a clean and dry maternity area, antiseptic dipping of the external umbilical cord, and timely feeding of colostrum, which are reported to reduce the incidence of umbilical infections [7, 17]. According to Van Camp et al., calves receiving colostrum soon after birth showed significantly reduced odds of developing external umbilical infections [18]. To date, the incidence of umbilical infections in Iranian dairy cattle farms is not well understood, and still, no comprehensive controlled farm study has been conducted. This study was conducted to determine the occurrence of umbilical infections in calves kept in range or semi-industrial husbandry systems. Also, the frequency of the infections in relation to different breeds, gender, and age of the calves was evaluated. Additionally, the results of the surgical treatments and outcomes were reported.

## 2. Materials and Methods

### 2.1. Animals and Data

The medical records of 550 calves admitted to the Veterinary Teaching Hospital, Faculty of Veterinary Medicine, Urmia University, Urmia, Iran, between March 1998 and July 2020 for treatment of umbilical masses were retrospectively analyzed. Of these, 250 cases of umbilical hernias and 62 cases with incomplete data records were excluded, while 238 calves with umbilical infections were included in the present study. The study procedures were reviewed and approved by the Animal Ethics Committee of Urmia University (Ref No. IR-UU-AEC-3/62), and written consent was obtained from the animal owners. The calves were managed under either range conditions or in smallholder semi-industrial (free stall intensive system) settings. Recorded data encompassed information on breed, sex, age, type of husbandry, clinical and surgical examination findings, as well as details on surgical techniques employed and their outcomes.

### 2.2. Clinical Examination and Systemic Medical Supportive Therapy

Upon admission, the calves underwent routine physical examinations, encompassing general health assessments. Diagnosis typically relied on comprehensive evaluations including historical data, clinical findings, and meticulous inspection of the umbilical region, particularly through deep abdominal palpation in lateral or semidorsal recumbency, focusing on specific characteristics of the umbilical mass. When necessary, transabdominal ultrasonography was employed to further delineate the contents of intra-abdominal umbilical remnants and verify the nature of potentially adhered structures, thereby validating the diagnosis. Subsequent management strategies involved a dual approach of conservative medical therapy and surgical intervention tailored to the final diagnosis.

Calves diagnosed with umbilical hernia and concurrent umbilical remnant infections were managed according to the protocols detailed in another study [19].

Reportedly, most umbilical abscesses responded to drainage and lavage of the abscess cavity with or without systemic antimicrobials [7]. However, these abscesses were treated with surgical resection, with the advantage of rapid elimination of all infected tissue [20]. In the present study, a drainage protocol was implemented for the treatment of umbilical abscesses in the affected calves. Shortly, an incision was made at the most ventral site of the fluctuant mass to facilitate drainage. Then, the abscess cavity was treated by dilute povidone iodine irrigation and curettage. Subsequent daily lavage with betadine or packing the cavity with betadine-soaked gauze was followed, and the packed gauze was gradually removed over time until complete healing. Additionally, calves received the following medical treatment regimen: intramuscular administration of procaine penicillin (22,000 IU/kg) and dihydrostreptomycin HCl (11 mg/kg), or oxytetracycline (10 mg/kg) once daily for three consecutive days [7]. Single intramuscular dose of vitamin AD3EC compound (3–6 mL/calf) was administered according to the manufacturer's instructions.

Calves diagnosed with umbilical cord remnants infections, such as enlarged umbilical stalk, chronic active omphalitis, and infections of remnants located in the intra-abdominal portion, received preoperative systemic medical supportive therapy. The medical support using the antimicrobials and vitamins, as mentioned earlier, was initiated prior to surgical intervention aiming to optimize the calf health status and enhance surgical outcomes.

### 2.3. Anesthesia and Surgical Techniques

To optimize hydration status, calves received supportive medication in the form of warm crystalloid isotonic solutions (such as normal saline or Ringer's solution) intravenously at a rate of 6–80 mL/kg/h prior to surgical intervention. Sedation, as needed, was achieved through intramuscular administration of xylazine hydrochloride at a dosage of 0.1 mg/kg body weight. Local anesthesia was administered using 2% lidocaine hydrochloride via infiltration in a ring block method. During the surgical procedure, the calves were placed on a tilt table in lateral or semidorsal recumbency position.

In the present study, calves presenting with hernia and concurrent infections of the external portion of the umbilical cord underwent treatment via the open herniorrhaphy technique [6] following the aforementioned preoperative medical supportive care therapy.

Calves diagnosed with chronic active omphalitis or enlarged umbilical stalk were treated by initial systemic antimicrobial therapy and vitamin administration as described earlier during their first admission and subsequently, during their second admission, the infected fibrous core or stalk was surgically removed via open ventral midline abdominal laparotomy [6] to prevent potential recurrence of the infection. Moreover, calves diagnosed with infections of the internal portion of umbilical remnants, including urachus abscesses, omphalophlebitis, and omphaloarteritis, either singularly or in combination, received the preoperative medical supportive therapy prior to surgical intervention. During their second admission, the infected remnants were surgically removed following abdominal laparotomy exploration through a ventral midline approach [6].

For calves presenting with omphalitis, typically aged between 4 and 10 days, conservative treatment was implemented. This included local umbilical therapy and administration of systemic antimicrobials, specifically: intramuscular administration of procaine penicillin (22,000 IU/kg) and dihydrostreptomycin HCl (11 mg/kg), or oxytetracycline (10 mg/kg) once daily for 3 to 5 consecutive days [7]. Dual intramuscular dose of vitamin AD3EC compound (3–6 mL/calf) were administered according to the manufacturer's instructions.

### 2.4. Postsurgical Management and Follow-Up Evaluations

Following surgical repair, calves received complementary antimicrobial and vitamin therapy as previously described. The surgical skin wound was irrigated with povidone iodine for 5–7 days to promote healing and prevent infection. Skin sutures were removed 14–16 days after surgery. Owners were advised to keep the calves in confined condition, box/stable, for 4 weeks following surgery. Follow-up information was gathered 2–4 weeks after surgery to assess for potential surgical complications such as suture abscesses, seromas, hematomas, wound dehiscence, hernia formation, abscess formation, and peritonitis.

### 2.5. Statistical Analysis

Descriptive statistics were computed using SPSS software (Version 27.0; SPSS Inc., Chicago, IL, USA) to analyze the relative frequency of umbilical infections in a retrospective hospital-based study. Only calves with complete information were included in the analysis. Simple statistical tools such as frequency distribution and percentage calculations were employed to calculate the frequency of umbilical infections among different groups or variables. The association between the occurrence of umbilical infection and sex, breed, and rearing system (range vs. semi-industrial) was evaluated using a 2 × 2 contingency table. Odds ratios (ORs) with 95% confidence intervals (CIs) were calculated, and Fisher's exact test was applied to determine statistical significance. A two-tailed *p*-value < 0.05 was considered significant.

## 3. Results

The prevalence of umbilical infections in the study population was determined to be 8.66%. A general descriptive analysis of the collected data from 238 calves revealed that 206 calves (86.55%) were affected solely by umbilical infections and 32 calves (13.45%) were affected by umbilical infections concurrently with umbilical hernias. The frequencies of umbilical infections in relation to breed, sex, and age in the affected calves (No. 238) are represented in Table 1. The prevalence of umbilical infections varied across different categories including 107 calves (44.96%) with umbilical abscesses, 110 calves (46.22%) with umbilical remnants infections, and 21 (8.82%) newborn calves with omphalitis. The distribution of infections between the external and intra-abdominal portions of the umbilicus was fairly balanced, with 52 calves (47.27%) affected externally and 58 calves (52.73%) affected internally.

**Figure 1:**
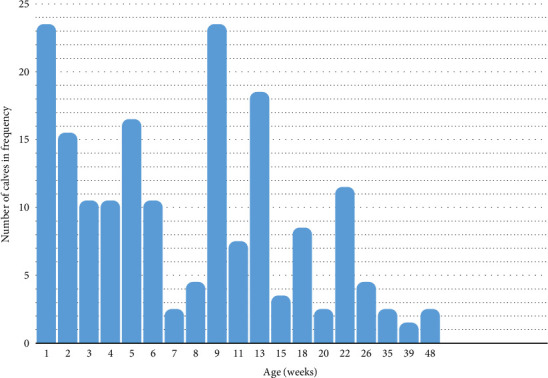
Frequency and distribution of umbilical infections in relation to age (in weeks) among 238 affected calves.

Figure 1 illustrates the distribution of umbilical infections among calves categorized by age. The age of the calves ranged from 1 to 48 weeks, with a median age recorded at 7 weeks. Over 49.1% of the calves were aged 6 weeks, and more than 80% were under 13 weeks old.  Table 2 provides detailed information on the frequency of anatomical umbilical infections categorized by breed, sex, and age among the affected calves. The age range of the calves included in the study extended from 3 to 330 days. The majority of calves affected by umbilical mass infections were kept under range management condition. Out of 238 calves with umbilical infections, 227 (95.42%) were reared under the range system, while only 11 (4.58%) were from the semi-industrial system. Fisher's exact test revealed a statistically significant association between the rearing system and the occurrence of umbilical infections (*p* < 0.001) indicating that calves under the range system had a significantly higher prevalence of umbilical infections compared to those in the semi-industrial system. However, the odds of umbilical infections were not significantly different between calves reared under the range system and those in the semi-industrial system (OR = 1.00; 95% CI: 0.51–1.97; *p* > 0.05).

In general, the frequency of calves affected by umbilical mass infections, with or without hernia, was almost equal between females and males with 115 calves (48.33%) and 123 calves (51.67%), respectively. Males had 15% lower odds of umbilical infection compared to females (OR = 0.85; 95% CI: 0.65–1.11); however, this difference was not statistically significant (*p*=0.21). There was no significant difference between the Holstein breed and other breeds in terms of the risk of umbilical infections (OR = 0.997; 95% CI 0.69–1.44; *p*=0.97).

In Table 2, the distribution of umbilical stalk infections among 210 affected calves is detailed by breed, sex, and age categories. The most prevalent type of umbilical mass infection observed was umbilical abscesses, accounting for 89 cases (42.38%), followed by chronic active omphalitis with 29 cases (13.81%), and infection of the urachus with 22 cases (10.48%).

The drainage protocol proved to be successful in treating umbilical abscesses in 95.51% of cases (85 out of 89), with only 4 cases (4.49%) requiring surgical intervention due to recurrence. Surgical extirpation during the second admission resulted in successful treatment without reported complications. Conservative local therapy followed by extirpation of the affected cord through linea alba laparotomy successfully treated 11 out of 14 cases of calves affected by fibrotic cord with abscess. Three of these cases were not admitted again for further surgical intervention. Sixteen calves with chronic active omphalitis and five with enlarged umbilical stalk responded effectively to the conservative systemic medical therapy. Moreover, surgical removal of the affected cord through midline laparotomy was successful in eight calves affected by enlarged umbilical stalk or fibrotic cord, with no reported complications.

**Figure 2:**
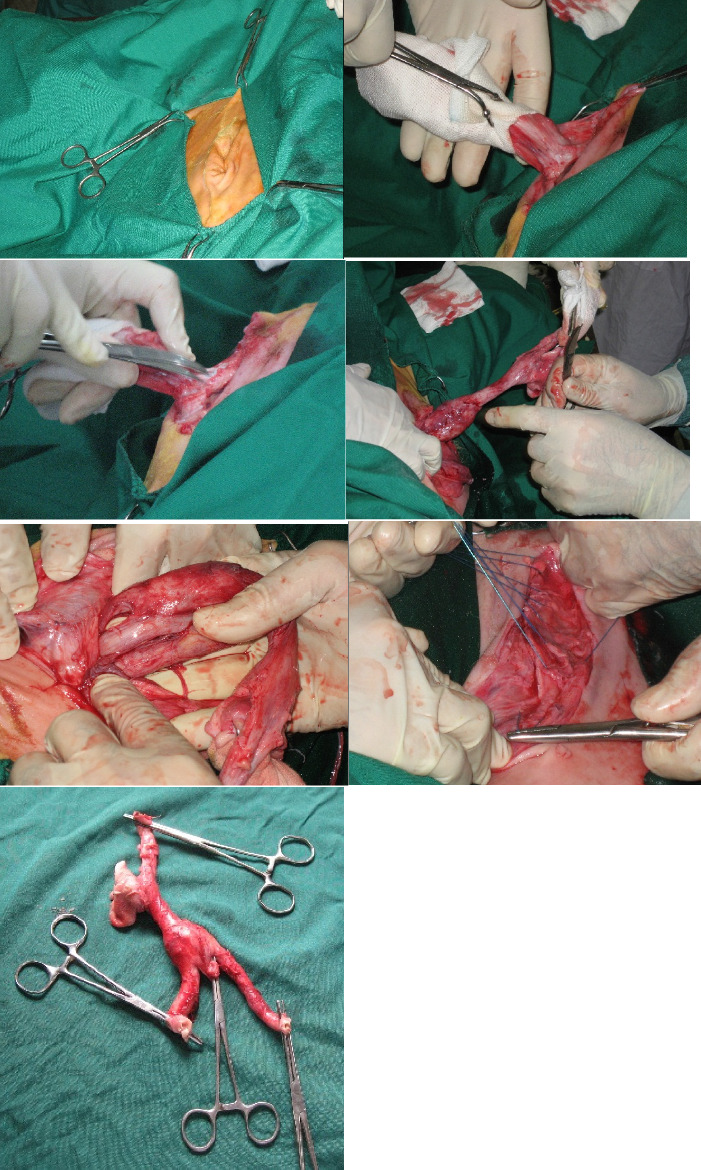
Intraoperative photographs of a calf with an externally normal-appearing umbilical cord, demonstrating internal infection involving the umbilical vein, paired umbilical arteries, and the urachus.

Figure 2 presents intraoperative photographs of a calf demonstrating infection of the internal umbilical structures, including the umbilical vein, paired umbilical arteries, and the urachus. Preoperative systemic and local medical supportive therapy administered over three consecutive days, followed by surgical extirpation of the infected internal remnant portion(s) of the umbilical cord via a ventral midline approach were successful in the treatment of the calves affected with the following conditions of urachus infection (No. 22), omphalophlebitis (No. 9), omphaloarteritis (No. 5), concurrent urachus infection with omphaloarteritis (No. 9), concurrent urachus infection with omphaloarteritis and omphalophlebitis (No. 6), concurrent urachus infection with omphalophlebitis (No. 2), and polyarthritis in the tarsal and/or carpal arthritis (No. 4). No post-operative complications were reported.

Conservative medical therapy proved to be highly successful in treating omphalitis in neonatal calves, with only one case reporting a subcutaneous abscess formation during follow-up. All affected calves were managed under range management practices. Regular antiseptic dipping of the external portion of the umbilical cord after birth was conducted in only one calf. Navel stalk ligation and cutting without antiseptics were performed in 3 out of 21 cases. In cases of active bleeding from umbilical arteries observed in six Holstein neonatal calves, appropriate ligation and local care led to full recovery without any complications. Additionally, a female native calf was diagnosed with congenital urethral agenesis and a persistent patent urachus, which necessitated a recommendation for culling.

## 4. Discussion

Umbilical disorders, including infections and hernias, rank as the third most frequent cause of disease in young calves under 3 months of age, typically occurring shortly after birth [1]. Navel ill, characterized by infection of the umbilicus and its associated structures, is a prevalent condition among newborn calves [21]. Recent studies in the United States have indicated that umbilical infections constitute the third most common illness in dairy calves, following digestive and respiratory diseases [18]. In the present study, the prevalence of umbilical infection was estimated at 8.66%. Previous reports reported a considerable range in the prevalence of umbilical infections in calves under 3 months of age. Svensson et al. documented a rate of 0.081 [22], while Fordyce et al. reported 9.0% [4]. Other studies reported higher rates including Virtala et al., 14.2% [1]; Hathaway et al., 29.9% [5]; and Steerforth and van Winden, 34.2% [23].

This long-term clinical descriptive study aimed to assess the prevalence of umbilical infections in calves reared in range or semi-intensive husbandry systems in Iran. The study found that umbilical abscesses and infections of umbilical remnants were the most commonly observed pathologies, whereas omphalitis was the least frequent form of navel ill among the studied calves. A notable finding of the study was the nearly equal frequency of remnant infections observed in the external versus intra-abdominal portions of the umbilicus. This finding emphasized a reevaluation of current disinfection practices aimed at preventing navel ill infections, particularly concerning the external portion of the umbilicus, given that routine navel dipping was not practiced in the range management observed in this study.

Reportedly, no significant differences were found between two groups of neonatal calves, with and without umbilical disinfectant dipping administered only once, regarding the incidence of external umbilical infections [18]. After parturition, the umbilical cord separates from the fetus, exposing the umbilical structures to the external environment. This exposure facilitates the entry of pathogens into the umbilical cord, potentially leading to umbilical infections [24]. Current recommendations suggest disinfecting, promoting drying, and facilitating healing of the navel by dipping 1–2 times daily for 3–4 days. However, one potential issue contributing to infection occurrence could be the short duration of dipping, given that the umbilical cord naturally dries within 1–8 days after birth in dairy calves [25]. Effective strategies to prevent umbilical infections in calves include using dry and clean bedding, meticulous hygiene in calving pens, administration of adequate volume of high-quality colostrum, and implementation of navel dipping protocols with appropriate antiseptics. Among these, ensuring the early ingestion of good-quality colostrum is paramount. Extensive evidence identifies timely colostrum intake as a critical preventative measure against navel ill, with consumption within the first 2 hours post-partum being strongly advocated [26, 27]. Van Camp et al. indicated that for every hour colostrum provision delayed after birth, the odds of developing an external umbilical infection increases by a factor of 1.15 [18].

Umbilical infections typically apparent in calves aged 2–6 weeks, attributed to their immature immune systems [17]. The present study results underscored that calves under 48 weeks (330 days) of age were at the risk of umbilical infections. However, a heightened risk was observed particularly in those in 6 weeks of age. Furthermore, calves aged 13 weeks faced a notably lower risk compared to younger counterparts, suggesting a diminishing risk as calves mature beyond 3 months.

In the present study, calves under the range system had a significantly higher prevalence of umbilical infections compared to those in the semi-industrial system, where formal after-birth umbilical cord therapeutic practices were less rigorously implemented in range compared to semi-intensive farms. In the present study, there was no significant difference in the risk of infection between female and male calves, suggesting that gender did not act as a predisposing matter for the occurrence of the infection. This finding was in agreement with previous research by Yanmaz et al. [28], which reported no significant association between gender and umbilical diseases [28]. Also, there was no significant difference between the Holstein and other breeds in the risk of umbilical infection; therefore, other factors such as the hygiene level of calving pens, bedding, and management practices could play a more critical role in the occurrence of the infection.

In the present study, the most prevalent infection affecting the external portion of the umbilical cord in affected calves was umbilical abscess, followed by chronic active omphalitis and urachus infection. This finding was consistent with a retrospective study on 126 Holstein calves from intensive farms, where umbilical abscess accounted for the highest prevalence at 44% among infections, followed by omphaloarteritis, omphalophlebitis, and urachus infection [29]. In a retrospective study involving 322 calves, omphalitis was identified as the most prevalent umbilical disease, followed by umbilical abscess, urachus infection, and omphalophlebitis [28]. Similarly, in a small-scale study evaluating the occurrence of umbilical infections in traditional rearing systems, omphalitis was reported as the most common condition, followed by umbilical abscess [27].

The results of the present study indicated that a significant majority (95.51%) of umbilical abscesses responded well to the drainage protocol. Surgical extirpation of affected tissues may be considered as a suitable option in cases of recurrence. Additionally, the drainage protocol followed by surgical extirpation was effective in treating calves with umbilical fibrotic cord abscesses. Conservative systemic medical therapy and surgical extirpation were both effective in managing chronic active omphalitis and enlarged umbilical stalk in calves. Surgical extirpation could be recommended in cases where cosmetic appearance is a concern. These findings were consistent with other investigations [6].

In the present study, when the intra-abdominal portion of umbilical structures was affected, urachus infection was the most common pathology observed, followed by omphalophlebitis as the second most infection, and omphaloarteritis, which was the least frequent pathology. The prevalence of umbilical cord remnant pathologies varies across studies. Omphalophlebitis has been reported as the 4th most common disease in calves, following diarrhea, respiratory diseases, and ringworm in calves aged 0–90 days [21].

Regarding the umbilical remnant pathologies identified in the present study, urachus infection was most commonly prevalent, which was followed by omphalophlebitis and omphaloarteritis. Consistent with our study, others reported urachus as the most frequently infected structure in cases of umbilical remnant infections in calves [7, 30]. Similarly, in one investigation on 76 cases with umbilical remnant infection, 52 cases with urachus infection were documented [28]. Also, Staller et al. reported 14 cases of urachus infection out of 32 cases with infected remnants [31]. However, Rodrigues et al. in 2010 noted a higher prevalence of patent urachus in 22 out of 44 presented umbilical disorders [32]. One study reported urachus infection to be less frequent than omphalophlebitis and omphaloarteritis [29]; however, most studies consistent with our results indicated that omphaloarteritis was typically the least common [7]. In one study on 76 infections, 24 cases of omphalophlebitis were documented with no cases of omphaloarteritis [28]. Staller et al. in 1995 reported 9 cases of omphalophlebitis and 6 cases of omphaloarteritis out of 32 cases with the umbilical remnants infection [31]. Furthermore, possible extension of urachus infection to the bladder must be considered during intervention [33]. However, in the present study, a complex infection involving the urachus along with the umbilical vein and arteries was observed in nearly 40% of cases with no bladder involvement. Surgical treatment proved to be a successful therapeutic modality for infections involving the urachus and/or the umbilical vessels.

Several umbilical disorders necessitate surgical intervention [2, 10, 14]. Prior to surgery, meticulous planning is crucial and ultrasonography can aid in selecting the optimal approach [11]. Accordingly, in the present study, surgery was performed upon confirmation of infection involving the intra-abdominal portion of the umbilical remnants.

Omphalitis was the least frequent infection in the present study, and conservative medical therapy was generally successful in treating omphalitis in affected neonates. Treatment typically involved appropriate broad-spectrum antimicrobial therapy along with topical antiseptics and supportive therapy. Surgical intervention was only required in rare cases where the infection progressed to abscess formation. Overall, good sanitation measures, boosting the immune system through proper colostrum feeding and regular care of the umbilical cord, can significantly reduce the incidence of omphalitis and other umbilical cord infections in neonatal calves [18, 27]. In our study, umbilical infections constituted more than 50% of navel ill cases significantly impacting the morbidity in calves managed in range and smallholder settings. The present study was a long-term clinical descriptive study that seems to be sound, from which the results could be extrapolated to calves raised in a range system; however, it had some limitations. In our regional range system, a controlled experiment is not a practical issue because there are many variations in the dam rations, setting calves' timely colostrum feeding and the farms health practices. One of the important limitations of the present study was the lack of accurate recording of colostrum feeding time in hours after birth. Hence, we could not calculate the association between the timely colostrum feeding with the incidence of umbilical infection. In general, timely colostrum feeding, careful and routine umbilical cord care with an antiseptic and keeping the calf in a clean, dry, well-ventilated area could be recommended as substantially effective measures in decreasing calf morbidity and mortality.

## 5. Conclusion

It was concluded that umbilical abscess was the most prevalent pathology observed in the external portion of the umbilicus, while omphalitis was the least frequent infection. In the intra-abdominal portion of the umbilicus, urachus abscessation was the most common pathology, followed by omphalophlebitis as the second most common, and omphaloarteritis as the least common. Umbilical infection was more prevalent in calves managed in range system compared to smallholder system. These infections were effectively managed through medical and/or surgical interventions, with surgery generally yielding a good prognosis in treating umbilical infections in calves.

## Figures and Tables

**Table 1:** Frequency of umbilical stalk or mass infections, with or without concurrent umbilical hernia, in relation to age, sex, and breed among 238 affected calves.

Age (days)	Sex	Breed	Umbilical cord remnant (s) infections no. (%)				
			Localized umbilical abscess	Enlarged stalk (external portion)	UVA infection (internal portion)	Omphalitis	Sum
≤ 30	Male	Holstein	9	10	9	11	39 (16.39)
		Native	1	—	—	1	2 (0.84)
		Others	—	—	—	—	0 (0.00)
	Female	Holstein	8	8	8	6	30 (12.61)
		Native	2	1	1	1	5 (2.10)
		Others	—	1	2	2	5 (2.10)

≥ 31 ≥ 60	Male	Holstein	14	6	4		24 (10.08)
		Native	1	1	—	—	2 (0.84)
		Others	—	—	1		1 (0.42)
	Female	Holstein	16	7	7		30 (12.61)
		Native	2	—	1	—	3 (1.26)
		Others	—	—	—	—	0 (0.00)

≥ 61 ≥ 90	Male	Holstein	7	4	7		18 (7.56)
		Native	2	—	—	—	2 (0.84)
		Others	1	3	—	—	4 (1.68)
	Female	Holstein	12	3	6		21 (8.82)
		Native	2	1	—	—	3 (1.26)
		Others	—	—	—	—	0 (0.00)

≥ 91 ≥ 336	Male	Holstein	16	5	3	—	24 (10.08)
		Native	5	—	1	—	6 (2.52)
		Others	—	—	1	—	1 (0.42)
	Female	Holstein	7	2	6		15 (6.30)
		Native	2	—	—	—	2 (0.84)
		Others	—	—	1	—	1 (0.42)

Sum	Male	Holstein	46	25	23	11	105 (44.12)
		Native	9	1	1	1	12 (5.04)
		Others	1	3	2	0	6 (2.52)
	Female	Holstein	43	20	27	6	96 (40.34)
		Native	8	2	2	1	13 (5.46)
		Others	0	1	3	2	6 (2.52)

Sum			107 (44.96%)	52 (21.85%)	58 (24.37%)	21 (8.82%)	238 (100%)

*Note:* Holstein: Holstein and Holstein–native cross. Others: Simental, Holstein–Simental cross, Simental–native cross. Enlarged stalk (external portion): fibrotic cords and chronic active omphalitis. UVA infection (internal portion): infection of the urachus, umbilical vein, and paired umbilical arteries in separate or concurrent.

**Table 2:** Frequency of umbilical stalk or mass infections in relation to age, sex, and breed considering anatomical involvement, in 210 affected calves.

Age (days)	Sex	Breed	Umbilical cord remnant (s) infections no. (%)											
			Localized umbilical abscess	Fibrotic cord with abscesses	Chronic active omphalitis	Urachus infection	Omphalophlebitis	Omphaloarteritis with urachus infection	Omphaloarteritis/Omphalophlebitis/Urachus infection	Omphaloarteritis	Urachus infections & omphalophlebitis	Omphalitis	Polyarthritis	Sum (%)
1–30	Male	Holstein	6	—	9	2	3	3	—	—	—	11	—	34
		Native	—	—	—	—	—	—	—	—	—	1	—	1
		Others	—	—	—	—	—	—	—	—	—	—	—	—
	Female	Holstein	6	2	3	1	3	—	2	1	1	6	—	25
		Native	2	—	1	—	—	—	—	1	—	1	—	5
		Others	—	—	—	—	—	—	1	—	1	2	—	4

31–60	Male	Holstein	13	—	5	1	—	—	2	1	—	—	2	24
		Native	1	—	1	—	—	—	—	—	—	—	—	2
		Others	—	—	—	1	—	—	—	—	—	—	—	1
	Female	Holstein	14	4	3	3	—	3	—	—	—	—	2	29
		Native	1	—	—	—	—	—	—	1	—	—	—	2
		Others	—	—	—	—	—	—	—	—	—	—	—	—

61–90	Male	Holstein	5	3	1	3	2	—	—	1	—	—	—	15
		Native	2	—	—	—	—	—	—	—	—	—	—	2
		Others	1	1	1	—	—	—	—	—	—	—	—	3
	Female	Holstein	10	2	—	3	—	3	—	—	—	—	—	18
		Native	1	—	1	—	—	—	—	—	—	—	—	2
		Others	—	—	—	—	—	—	—	—	—	—	—	—

91–336	Male	Holstein	16	1	3	3	—	—	—	—	—	—	—	23
		Native	4	—	—	1	—	—	—	—	—	—	—	5
		Others	—	—	—	—	—	—	—	—	—	—	—	—
	Female	Holstein	6	1	1	4	1	—	—	—	—	—	—	13
		Native	1	—	—	—	—	—	—	—	—	—	—	1
		Others	—	—	—	—	—	—	1	—	—	—	—	1

Sum	Male	Holstein	40	4	18	9	5	3	2	2	0	11	2	96 (45.71)
		Native	7	0	1	1	0	0	0	0	0	1	0	10 (4.76)
		Others	1	1	1	1	0	0	0	0	0	0	0	4 (1.90)
	Female	Holstein	36	9	7	11	4	6	2	1	1	6	2	85 (40.48)
		Native	5	0	2	0	0	0	0	2	0	1	0	10 (4.76)
		Others	0	0	0	0	0	0	2	0	1	2	0	5 (2.38)

Sum: No. (%)			89 (42.38)	14 (6.67)	29 (13.81)	22 (10.48)	9 (4.29)	9 (4.29)	6 (2.86)	5 (2.38)	2 (0.95)	21 (10.00)	4 (1.90)	210 (100)

*Note:* Holstein: Holstein and Holstein–native cross. Others: Simmental, Holstein–Simmental cross, Simmental–native cross.

## Data Availability

The data that support this study are available from the corresponding author upon request.
